# Brine Shrimp Feeding Contributes to Fast Growth and Enhanced Immune Capacity of Reattached Polyps of Scleractinian Coral *Pocillopora damicornis*

**DOI:** 10.3390/ani15223318

**Published:** 2025-11-17

**Authors:** Haifeng Huang, Yi Wang, Zhaoqun Liu

**Affiliations:** 1 Nansha Islands Coral Reef Ecosystem National Observation and Research Station, Guangzhou 510310, China; 2 School of Marine Biology and Fisheries, Hainan University, Haikou 570228, China; alvinhaifeng@163.com (H.H.); wangyi940317@163.com (Y.W.)

**Keywords:** *Pocillopora damicornis*, polyp reattachment, brine shrimp feeding, growth, immunity

## Abstract

Reef restoration is the major way to compensate the loss of scleractinian corals, which requires huge amounts of transplantation donors. Previous study revealed that some species of corals can conduct polyp bailout and reattachment under environmental stress, which contributes to the living of coral communities and offer a novel way to produce numerous coral colonies for reef restoration. In the present study, physiological and transcriptomic approaches were conducted to illustrate the effects and molecular mechanisms of brine shrimp feeding on the newly attached polyps of coral *Poccillopora damicornis*. It was observed that brine shrimp feeding significantly prompted the growth of reattached polyps by elevating polyp diameter, number of new polyps, weight of the calcified skeleton, symbiont density, chlorophyll *a + c_2_* content and Ea values. Transcriptomic analysis also inferred that signaling pathways responsive for energy metabolism, cell growth and biomineralization were dramatically activated. Furthermore, brine shrimp feeding enhanced the immunity of the reattached polyps by suppressing caspase-3 activation level and elevating antioxidant capacity. These results collectively reveals the influence and detailed molecular mechanisms of brine shrimp feeding on the growth of newly reattached coral polyps, which shed light on the potential application of such methods in the cultivation of coral transplantation donors.

## Simple Summary

The present study investigates how brine shrimp feeding contributes to the growth of newly reattached polyps of scleractinian coral *Poccillopora damicornis*. It was illustrated that polyp diameter, number of new polyps, weight of the calcified skeleton, symbiont density, chlorophyll *a + c_2_* content and Ea values increased significantly after 60 days of brine shrimp feeding, and the immune capacity of the reattached polyps were also obviously elevated. These findings illuminate the molecular mechanisms mediating fast growth of coral polyps upon brine shrimp feeding, and shed light on the potential application of such methods in the cultivation of transplantation donors for reef restoration.

## 1. Introduction

Coral bleaching occurs worldwide with increasing frequencies and intensities, which is caused by the stress response of stony coral to environmental change, especially increased sea surface temperature [1]. The coral reef restoration is the major way to compensate the loss of scleractinian coral caused by heat stress [2,3,4]. Based on the current reports, the main technologies for coral reef ecological restoration include sexual cultivation, asexual cultivation and artificial transplantation of corals [5]. Thus, understanding the reproduction process of scleractinian corals is of great importance since the coral reef restoration requires a huge amount of new coral colonies [6,7]. Scleractinian corals perform both sexual and asexual reproduction [6,7]. Even though only 10~13% of scleractinian corals conduct asexual reproduction, the asexual cultivation technology is the preferentially propagation method to reproduce coral donors via fragmentation. Generally, coral nubbins are collected from healthy corals and cultivated artificially in gardening nursery, in which way huge amounts of donors can be obtained within several months. In addition, corals rely on such artificial cultivation conditions or field cultivation conditions to promote their growth rate, so that they can grow to a suitable size, and to meet the required size and number for reef restoration [8]. The asexual cultivation of reef-building corals can be successfully conducted under suitable and artificially controlled conditions, where various environmental factors such as light, temperature, and nutrient salts are maintained within the optimal range for corals, enabling the corals to achieve their optimal physiological state. This not only significantly enhances the growth rate of corals but also greatly improves their survival rate after transplantation [9,10,11,12]. However, the asexual cultivation of reef-building corals has several drawbacks, including high cost, operational difficulty, and potential damage to healthy corals. According to a report from The Joint Research Centre, European Emission, it was estimated that restoring just 10% of the world’s degraded reefs would cost at least USD 1 billion (around EUR 926 million), which is almost four times the total investment made over the past decade. Even when restoration projects succeed, their benefits are often short-lived. Also, coral donors obtained from asexual reproduction inherently limits the proliferation of different genotypes and hence resilience [8,9,10]. Therefore, developing and promoting new asexual cultivation techniques for reef-building corals is crucial and imperative for the ecological restoration of coral reefs [13,14,15]. In recent years, an asexual reproduction process called “polyp bailout” has been documented in scleractinian corals, which might be a novel and effective way to produce coral transplantation donors [16,17,18,19].

Polyp bailout refers to the release and reattachment of polyps once parent corals face an unfavorable environmental conditions, which is a stress response phenomenon and a survival strategy of scleractinian corals [20,21]. Polyp bailout is characterized by the dissociation of coral colonies via coenosarc degradation and the detachment of polyps from the calcareous skeletons [21]. Such process might represent an example of reverse development (Piraino et al. 2004) in which a detached polyp undergoes complete tissue and cell rearrangement with loss of the compartment structure present in the primary polyp [22,23]. For example, the tree coral (*Astroides calycularis*) actively releases polyps under low-nutrient conditions. These polyps can survive in the environment for 2–3 months and eventually reattach and grow into new coral colonies in suitable marine areas. Caribbean octocoral (*Eunicea flexuosa*) releases healthy polyps before irreversible physiological damage occurs across the entire colony when faced with thermal and desiccation stress, thereby preserving its genetic information [24,25]. Moreover, as a form of asexual reproduction, polyp bailout is also an effective supplement to coral sexual reproduction, helping coral populations to maintain their numbers under fluctuating environmental conditions. Thus, polyp bailouts represent a unique adaptive mechanism by which corals respond to oceanic environmental changes such as heat stress, playing a key role in population maintenance, population dispersal, and genetic information transfer. Furthermore, based on our estimation, by leveraging the polyp bailout and reattachment mechanisms, it is possible to cultivate the same number of coral transplant donors with fewer (decreased about 80%) coral parents comparing with traditional fragmentation method, thereby significantly improving the efficiency of coral reef ecological restoration.

In order to enhance the efficiency of coral restoration, it is significant to accelerate the growth of transplantation donors, in which the “polyp bailout and reattachment” method could play an important role. Corals are heterotrophic organisms; heterotrophic feeding is essential for them if they are to avoid nutrient deficiency. Corals exhibit several modes of feeding and obtain energy from multiple sources [26,27]. In addition to obtaining nutrients from symbiotic algae, corals can feed on marine plankton, which provide essential amino acids and other nutrients not obtainable from symbiotic algae [28,29,30]. Numerous studies have also reported that many species of corals are also active heterotrophs, ingesting bacteria, particulate organic matter, and even dissolved nutrients from water and mesozooplankton. Heterotrophy accounts for up to 66% of the fixed carbon incorporated into the skeleton of a coral and meets 15–35% of the daily metabolic requirements, contributing to the metabolic activity associated with Symbiodiniaceae [31,32,33]. Regarding the feeding habits of the coral *Montastraea cavernosa*, the larvae of copepods, amphipods, nemerteans, turbellarians, polychaetes, nematodes, appendicularians, salps, decapods, and cirripedes have been discovered in the coral body and planktonic algae [34]. Studies have suggested that corals are polytrophic in nature [35]. However, most of the previous studies generally focused on the feeding behavior of adult corals, while process in the early life of corals was neglected.

Polyp bailout is an adaptive environmental mechanism which is shared by several coral families, including cup corals (*Pocilloporidae*), staghorn corals (*Acroporidae*), tree corals (*Dendrophylliidae*), black corals *(Antipathidae*), and caryophyllia corals (*Caryophylliidae*), with the *Pocilloporidae* family being the most frequently described [24,25,36,37,38]. And, our previous research proved that heat treatments could induce significant polyp bailout in *P. damicornis* within 7 days. Therefore, the present study, using *P. damicornis* as research subject, investigates how brine shrimp feeding contributes to the growth of newly reattached polyps of *P. damicornis*, aiming to (1) reveal the influence of brine shrimp feeding on the growth of newly reattached coral polyps; (2) explore the molecular mechanisms mediating fast growth of coral polyps upon brine shrimp feeding; and (3) discuss the potential application of such methods in the cultivation of coral transplantation donors.

## 2. Materials and Methods

### 2.1. Coral Polyp Bailout and Reattachment

Six *Pocillopora damicornis* colonies (>5 m distance) in diameter of about 15 cm (almost the same size) were collected from a fringing reef in Sanya (18°24′2″ N, 109°37′54″ E), Hainan Province, China at the depth of ~3 m in October 2024. Before the experiment, each parent coral was cultured in a 40 L flow-through tank with natural seawater (temperature: 28 °C; salinity: 35), which was illuminated with a set of LED light bulbs (~500 μmol photons m^−2^ s^−1^ of light intensity) in a 12 h/12 h light-dark cycling. These parent corals were acclimatized for two weeks in the laboratory conditions [39].

The seawater temperature was generally controlled using a water tank heater (EHEIM 3612, Deizisau, Germany) to increase by 1 °C every 24 h from 28 °C to 31 °C and remained stable at 31 °C until the end of the experiment to induce the bailout of coral polyps. The entire process of polyp extension, polyp contraction, coenosarc dissociation, and eventual polyp bailout under high temperatures was observed with a stereomicroscope (Cnoptec, Chongqing, China). Furthermore, a total of 500 intact bailed-out polyps were randomly collected from the six coral colonies, and transferred to a water tank, which contained sea water of the same environmental conditions. Also, ten rocks collected from the reef area with algae and biofilm on them, were placed in the tank as the substrate for polyp reattachment. After one week (7 days), about 200 polyps were successfully reattached on the substrates, which were employed in the following feeding experiment.

### 2.2. Brine Shrimp Feeding, Observation and Sample Collection

**Figure 1:**
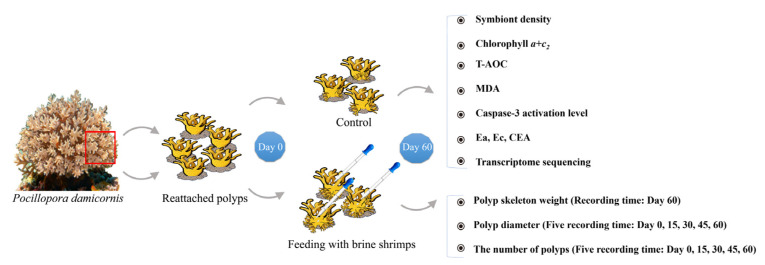
Flowchart showing the experimental design of the present study.

*Artemia* sp. is a natural food with high protein content, especially amino acid. According to previous study, the newly hatched *Artemia* sp. contains about 50.6% protein, 25.7% carbohydrate, 14.2% fat, 9.4% ash and the energy value of 18.97 KJ g^−1^ [40]. In the present study, a total of 100 reattached polyps were employed in the feeding experiment, while the same numbers of polyps were employed in the control group. As for the polyps in the feeding group, brine shrimps (*Artemia salina*) were fed to the reattached polyps on a daily basis, and the feeding treatments lasted for two months (60 days). Polyps were observed to feed on these shrimps, and this process usually lasted for 20~60 min each time. Polyps in both control and feeding groups were cultivated in a circulation tank and illuminated with a set of LED light bulbs (~500 μmol photons m^−2^ s^−1^ of light intensity) in a 12 h/12 h light-dark cycling. During the experiment, the polyp diameters were recorded by the stereomicroscope of LEICA M205 C (LEICA, Wetzlar, Germany), and the number of polyps were observed and calculated every day. At the end of the experiment (60th day), the calcified skeletons of all the live coral colonies (grown from the reattached polyps) were sampled after removing the soft tissues, and the weight of the calcified skeleton of each coral colony was determined. Meanwhile, coral soft tissues and algal symbionts were employed in transcriptomic and physiological assays (Figure 1).

### 2.3. Determination of Algal Symbiont Density and Chlorophyll Content

The algal symbiont density and chlorophyll content in corals were determined according to our previous studies with minor modifications [41,42]. Briefly, a Waterpik water jet with approximately 10 mL of pre-cooled PBS (pH 7.4, 4 °C) was applied to strip the coral tissue from the skeleton. The homogenate was then centrifuged at 12,000× *g*, 4 °C for 15 min to separate coral tissue supernatants and the pelleted symbionts. The symbionts from 1 mL of the homogenates were harvested through the centrifugation at 5000× *g*, 4 °C, 10 min, fixed in 4% formaldehyde solution, and counted with Sedgwick-Rafter counting chamber (Phycotech, St. Joseph, MI, USA). The symbiont density in corals was calculated by the normalization of symbiont number against the coral surface area obtained using a three-dimensional laser scanner (ZT-100, Zuote, Yongkang, China) [43], and were presented as cells cm^−2^. Furthermore, the symbionts harvested from 2 mL of the homogenate were resuspended in 2 mL of 100% acetone for 48 h to extract chlorophyll *a* + *c_2_*. The OD_630_ and OD_663_ of the extracts were measured with the spectrophotometer (UV-100, MAPADA, Shanghai, China) and acetone as the blank control to compute the total chlorophyll *a + c_2_* content through the equations of Jeffrey et al. [44]. The total content was further divided by the symbiont number to yield the chlorophyll content (pg cell^−1^).

### 2.4. Detection of Caspase-3 Activation Level and Antioxidant Capacity

The coral tissue supernatants obtained previously was employed for the determination of caspase-3 activation level. Meanwhile, the harvested symbionts from 1 mL of the coral tissue homogenates were resuspended in 1mL of PBS and homogenized using glass beads (0.5 mm diameter ceramic beads), and a bead homogenizer (Bioprep-24, Allsheng Instruments Co., Ltd., Hangzhou, China) at the setting of 6 m s^−1^ and duration of 1 min until all algal cells were found broken under the microscope. The homogenate was then centrifuged at 12,000× *g* 4 °C for 10 min to obtain the supernatant.

The caspase-3 activities in the homogenate supernatants of corals and their algal symbionts were measured by Caspase-3 Colorimetric Assay Kit (KeyGEN, Nanjing, China) according to the instruction. In this study, the caspase-3 activation level in corals was defined as “A405 values of coral from the feeding group (n = 5)/A405 values of coral from the control group (n = 5)”. The caspase-3 activation level in the algal symbiont was defined as “A405 values of algal symbiont from the feeding group (n = 5)/A405 values of algal symbiont in the control group (n = 5)”. In addition, the total antioxidant capacity (T-AOC) in corals were measured with test kits (A105, Nanjing Jiancheng Bioengineering Institute, Nanjing, China). T-AOC (U mg^−1^) was determined and illustrated as “units per milligram of tissue protein” [45].

### 2.5. Energy Metabolism in Corals

The energy availability (Ea) of corals were measured and calculated. Briefly, 250 μL of chloroform, 250 μL of methanol and 125 μL of Milli-Q water (STAR-VF-10, LIDING, Shanghai, China) were added into 150 μL of the tissue supernatants from corals. The mixture was centrifuged at 1000× *g* for 5 min to obtain the aqueous layer, and then 100 μL of extract suspension were added into 500 μL of H_2_SO_4_, and charred at 200 °C for 15 min. Finally, 1.5 mL of Milli-Q water was added to the above mixture, and total lipid content was measured at 375 nm with tripalmitin as standard. Total carbohydrate content was determined using the method with a few modifications [46]. In brief, 150 μL of the supernatant was added into 50 μL of trichloroacetic acid and held at −20 °C for 10 min, followed by a centrifugation at 1000× *g* for 10 min. Fifty-microliter supernatant was added into 100 μL of 5% phenol and 500 μL of H_2_SO_4_. After the mixture was incubated at 26 °C for 30 min, its absorbance at 492 nm was measured with glucose solution (0, 0.08, 0.16, 0.24 and 0.32 mg mL^−1^) as standard. Total protein content in the supernatants (of coral tissues and bailed-out polyps prepared in the above section) was quantified using BCA Protein Assay kit (C503021-0500, Sangon Biotech, Shanghai, China). Ea was calculated based on the carbohydrate, lipid, and protein content [47,48]. The Ea values in parent corals were normalized against the coral surface area and were presented as J cm^−2^, and the Ea values in the bailed-out polyps were normalized against polyp number (J polyp^−1^). In addition, mitochondrial electron transport system activity (ETSA) was measured. Fifty microliters of the supernatant was added to 150 μL of buffered substrate solution (0.13 M Tris-Cl, 0.3% Triton X-100, pH 8.5) and 50 μL NAD(P)H solution (1.7 mM NADH and 250 μM NADPH). The reaction was started by adding 50 μL of 8 mM p-iodonitrotetrazolium (INT), and the absorbance was measured kinetically at 26 °C for 10 min. Ec was converted according to the ETSA, and CEA was calculated through the following formula: CEA = Ea/Ec.

### 2.6. RNA Extraction and Transcriptome Sequencing

Total RNA was isolated from corals using the TRIzol reagent (Invitrogen, Waltham, MA, USA) with bead-beating homogenization (0.5 mm ceramic beads; Bioprep-24, Allsheng, Hangzhou, China), followed by chloroform extraction, isopropanol precipitation, and column purification (Zymo Direct-zolTM RNA MiniPrep Kit, R2052, ZYMO Research, Owen, WI, USA). RNA integrity and concentration were assessed using an Agilent 2100 Bioanalyzer (RNA 6000 Nano LabChip Kit; Agilent Technologies, Mulgrave, Australia) and a NanoDrop ND-2000 spectrophotometer (Thermo Scientific, Waltham, MA, USA), respectively. The paired-end transcriptome libraries (2 × 150 bp) were successfully constructed and sequenced on the BGISEQ-500 platform (BGI, Shenzhen, China), and two samples were excluded due to library preparation failures. Five replicates (each replicate refers to one coral colony grown from one reattached polyp) were performed for the transcriptomic assay. Following sequencing, the raw data were processed by filtering out contaminants, adapters, and low-quality sequences. The data quality was further monitored by analyzing the base distribution and base composition. The *P. damicornis* genome sequence and annotations (http://reefgenomics.org/, accessed on 25 July 2025) were used as references. Clean paired-end reads were mapped to the coral reference genome using HISAT2, and differential expression genes (DEGs) across different stages were identified using StringTie (v3.0.3) and DESeq2 (v1.48.2) (FDR < 0.05). Unmapped paired-end reads were merged and further assembled into transcripts using Trinity (v2.15.2) [49]. The assembled transcripts were subjected to BLASTX (v2.17.0) searches (value = 1 × 10^5^, max_target_seqs = 1) against protein databases of *Symbiodinium microadriatium*, *Breviolum minutum*, *Cladocopium goreaui*, and *Fugacium kawagutii* to obtain their genomic sequences. These aligned transcripts were considered symbiotic Symbiodiniaceae transcripts and were used as references for differential gene expression analysis across stages using the HISAT2-StringTie-DESeq2 pipeline (v2.17.0) [50,51]. Since the coral colonies employed in transcriptomic sequencing were from only six parent corals, the parent genotype was modeled as a random effect in this study.

### 2.7. Gene Co-Expression Network Analysis

To explore the coordinated expression patterns among samples, a correlation-based and hierarchical clustering approach was applied for gene co-expression network analysis. First, the transcriptomic expression matrix was normalized, and low-expression genes were filtered out. Pairwise Pearson correlation coefficients were then calculated among all genes to obtain a gene–gene correlation matrix. This matrix was converted into a distance matrix (1 − |r|) and subjected to hierarchical clustering using Ward’s minimum variance method (Ward.D2). To define the module boundaries from the clustering dendrogram, the dynamic tree cut method implemented in the R package DynamicTreeCut (v1.63) was applied. Modules with highly similar eigengene expression patterns (Pearson’s r > 0.75) were subsequently merged to ensure biological consistency. For each module, a module eigengene (ME) representing the first principal component of all gene expression profiles within the module was calculated. The correlation between each gene and its corresponding module eigengene (kME) was computed to quantify the gene’s representativeness of the module. The distributions of MEs and phenotypic traits were examined for normality using the Shapiro–Wilk test. As both variables approximately followed normal distributions, Pearson’s correlation coefficient was used to assess linear associations. To enhance biological consistency, modules with highly similar eigengenes (Pearson’s r > 0.75) were subsequently merged. For each module–trait pair, Pearson correlations and their corresponding p values were calculated and adjusted using the Benjamini–Hochberg false discovery rate (FDR) method. Correlations with adjusted *p* values < 0.05 were considered statistically significant. Relationships among modules and overall expression trends were visualized using heatmaps and eigengene clustering dendrograms. Genes within each module, along with their assigned module colors and kME values, were exported for subsequent functional annotation and enrichment analyses. Module-specific node and edge information was exported for visualization in Cytoscape. All analyses were performed in R (v4.4.2), and network visualization was carried out in Cytoscape (v3.9.1).

### 2.8. Statistical Analysis

Five biological replicates were conducted for the determination of each physiological parameter. Physiological data were first checked for the normality and homogeneity of variance with Shapiro–Wilk test and Levene’s test, respectively. Data that met the requirements were subjected to Student’s *t*-test to determine significant differences between the control and feeding groups, while the physiological data which did not meet normality or homogeneity of variance was subjected to a nonparametric test (Mann–Whitney U test). These analyses were performed using SPSS 20.0, and *p* < 0.05 was set as statistically significant. Principal component analysis (PCA) was performed on the physiological parameters of corals and their symbionts using Ggbiplot package in R 3.5.1 software.

## 3. Results

### 3.1. The Growth of Reattached Polyps After Brine Shrimp Feeding

**Figure 2:**
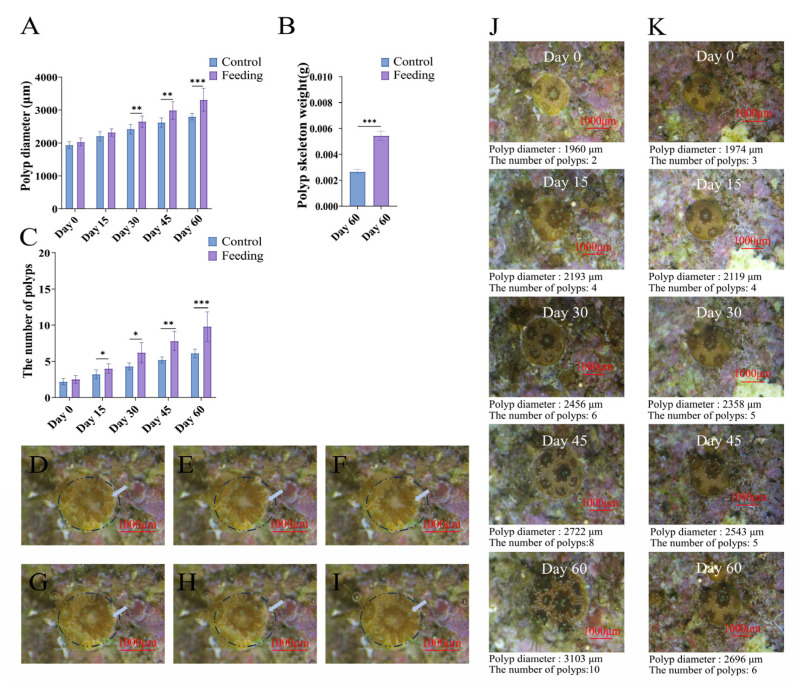
Changes in polyp diameter (**A**), numbers of polyps (**B**), and weight of calcified skeleton (**C**) after feeding. (**D**–**I**) show the step-by-step capture and ingestion of a brine shrimp: (**D**) brine shrimp near the mouth of polyp, (**E**) brine shrimp is captured by the mouth, (**F**) polyp is ingesting the brine shrimp; (**G**) the brine shrimp is almost ingested, (**H**): the brine shrimp is completely ingested; (**I**): the polyp at normal status; (**J**) and (**K**) show the growth of reattached polyps in the control (**J**) and feeding (**K**) groups on the 0, 15th, 30th, 45th and 60th day after brine shrimp feeding. “*” refers to *p* < 0.05, “**” refers to *p* < 0.01, and “***” refers to *p* < 0.001.

In the present study, the brine shrimp feeding (Figure 2D–I showed the detailed process) caused significantly accelerated growth of the reattached coral polyps. On the 30th day after feeding treatment, the average diameter of corals in the control group was 2419.40 ± 137.26 μm, which was significantly lower than that in the feeding group (2650.70 ± 163.42 μm) (*p* < 0.05, Figure 2A). By the end of the experiment (60th day), the average diameter of corals in the feeding group (3308.50 ± 331.11 μm) was also obviously higher than that in the control group (2797.9 ± 89.98 μm) (*p* < 0.05, Figure 2A). In addition, the number of new polyps of corals was recorded after feeding treatment, and the significant difference was observed after feeding for 14 days (Figure 2J,K). On the 15th day, the number of polyps in the control group was 3.20 ± 0.60, while that in the feeding group was 4.00 ± 0.63 (*p* < 0.05, Figure 2B). On the 60th day, the number of polyps in the feeding group (9.80 ± 1.93) was significantly higher than that in the control group (6.10 ± 0.53) (*p* < 0.05, Figure 2B). Moreover, the weight of calcified skeleton of corals after feeding for 60 days was determined. The average skeleton weight in the feeding group was 0.0054 ± 0.00036 g, which was significantly higher than that in the control group (0.00267 ± 0.0002 g) (*p* < 0.05, Figure 2C).

### 3.2. Energy Metabolism of Reattached Polyps After Brine Shrimp Feeding

**Figure 3:**
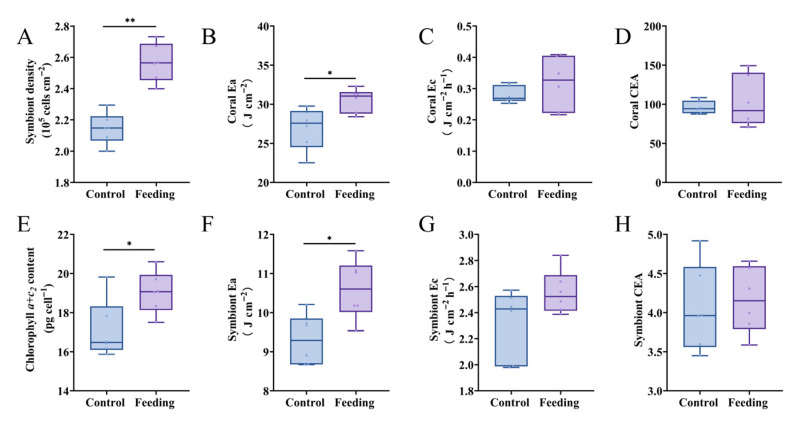
Changes in physiological parameters including symbiont density (**A**), chlorophyll *a + c_2_* content (**E**), Ea (**B**,**F**), Ec (**C**,**G**), and CEA (**D**,**H**) in the coral hosts and their algal symbionts after brine shrimp feeding. “*” refers to *p* < 0.05, “**” refers to *p* < 0.01.

To explore the effects of brine shrimp feeding on the growth of reattached polyps, parameters (algal symbiont density, chlorophyll *a + c_2_* content, Ea, Ec and CEA) related to energy metabolism in corals were tested. The symbiont density in the control group was 2.15 ± 0.10 × 10^5^ cells cm^−2^, which was obviously lower than that in the feeding group (2.57 ± 0.12 × 10^5^ cells cm^−2^) (*p* < 0.05, Figure 3A). The chlorophyll *a + c_2_* content in the control group was 17.11 ± 1.49 pg cell^−1^, which was obviously lower than that in the control group (19.05 ± 1.07 pg cell^−1^) (*p* < 0.05, Figure 3E). In addition, Ea value in coral host and their agal symbionts were 30.51 ± 1.52 J cm^−2^ and 10.60 ± 0.76 J cm^−2^, which were significantly higher than those in the control group, respectively (26.92 ± 2.67 J cm^−2^ and 9.31 ± 0.65 J cm^−2^) (*p* < 0.05, Figure 3B,F). As for Ec and CEA values, no significant difference was observed between control and feeding groups in both coral hosts and their agal symbionts (*p* > 0.05, Figure 3C,D,G,H).

### 3.3. Changes in Immune Capacity After Brine Shrimp Feeding

**Figure 4:**
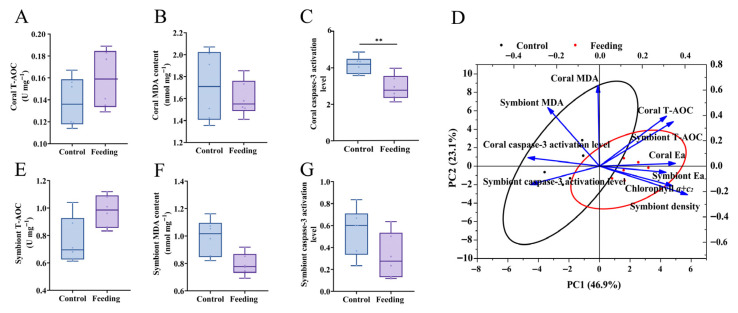
Changes of immune-related parameters including T-AOC (**A**,**E**), MDA content (**B**,**F**), and caspase-3 activation level (**C**,**G**) in the coral hosts and their algal symbionts after brine shrimp feeding. (**D**) showed results of PCA. “**” refers to *p* < 0.01.

To further reveal the contribution of brine shrimp feeding to reattached coral polyps, parameters relative to immune capacity, including T-AOC, MDA contents and caspase-3 activation level, were determined in this study. It was found that the caspase-3 activation level of coral hosts in the feeding group was obviously lower than that in the control group (*p* < 0.05, Figure 4C). However, T-AOC and MDA contents in both coral hosts (Figure 4A,B) and their algal symbionts (Figure 4E,F) showed no significant differences between control and feeding groups (*p* > 0.05).

Furthermore, the PCA was conducted to reveal the major physiological parameters that influence the growth and immunity in corals after brine shrimp feeding. The PCA biplot of the significantly changed physiological parameters illustrated 70.0% variability, with PC1 contributing 46.9% and PC2 contributing 23.1%. PC1 was correlated with symbiont density and chlorophyll *a + c_2_* content, while PC2 was correlated with coral and symbiont MDA contents. The orientation and length of each parameter revealed that coral MDA and symbiont density might contribute to the separation between the control and feeding groups (Figure 4D). Although coral’s MDA contents did not differ significantly between groups, it still contributed strongly to multivariate separation (loading value of 0.94 on PC2), possibly owing to their synergy with other variables.

### 3.4. Differentially Expressed Gene and GO Enrichment

**Figure 5:**
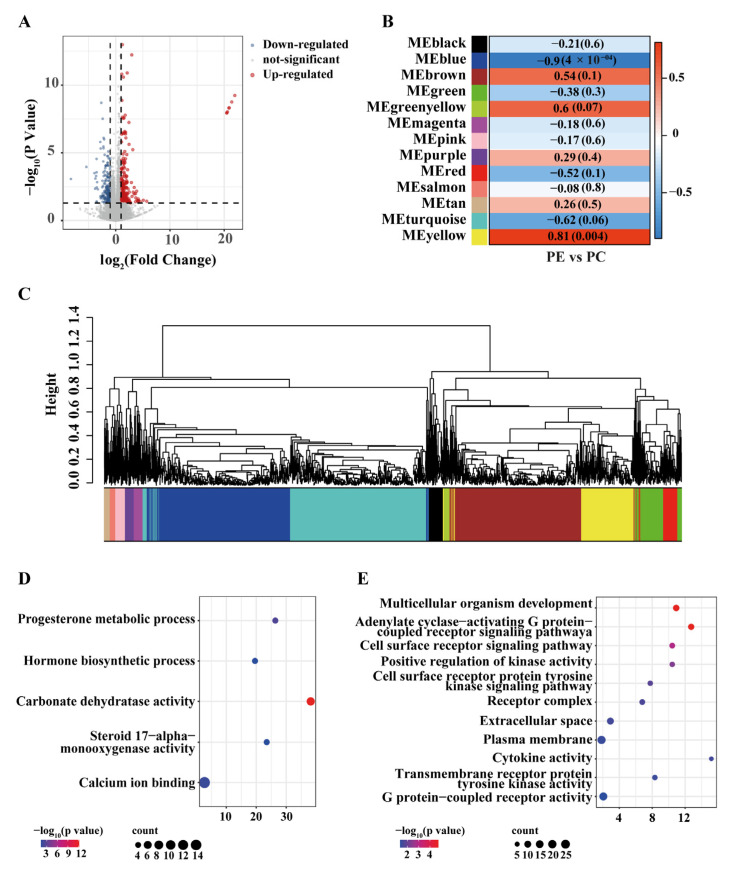
Overview of transcriptomic profiling and functional enrichment analysis. (**A**) Volcano plot of differentially expressed genes (DEGs). (**B**) Correlation heatmap of module eigengenes (MEs). (**C**) Gene clustering and module identification based on co-expression analysis. (**D**) GO enrichment analysis of significantly expressed genes in the control group. (**E**) GO enrichment analysis of significantly expressed genes in the feeding group.

Transcriptome sequencing was performed on hydroid samples from the control group and the feeding group, with five technical replicates per group. The gene mapping ratios of all samples were shown in Table 1. A total of 19,397 genes were detected in the transcriptome (Figure 5A). According to the screening criteria of |log2(Feeding/Control)| > 1 and q value < 0.05, 450 significantly differentially expressed genes (DEGs) were identified. Among them, 230 genes were significantly upregulated in the control group (log_2_ control group > 0), and 220 genes were significantly upregulated in the feeding group (log_2_ feeding group < 0).

To further investigate the functional differences in gene expression between the two groups, GO enrichment analyses were performed separately on the significant DEGs of each group. The results showed that in the control group, DEGs were significantly enriched in progesterone metabolic process (GO:0042448), hormone biosynthetic process (GO:0042446), carbonate dehydratase activity (GO:0004089), steroid 17-alpha-monooxygenase activity (GO:0004508), and calcium ion binding (GO:0005509) (Figure 5A, *p* < 0.05). In contrast, DEGs in the feeding group were significantly enriched in multicellular organism development (GO:0007275), adenylate cyclase-activating G protein-coupled receptor signaling pathway (GO:0007189), cell surface receptor signaling pathway (GO:0007166), positive regulation of kinase activity (GO:0033674), cell surface receptor protein tyrosine kinase signaling pathway (GO:0007169), extracellular space (GO:0005615), cytokine activity (GO:0005125), transmembrane receptor protein tyrosine kinase activity (GO:0004714), and G protein-coupled receptor activity (GO:0004930) (Figure 5B).

### 3.5. Co-Expression Network and Functional Module

To further investigate the co-expression patterns among differentially expressed genes (DEGs) as well as their regulatory relationships and biological functions, a co-expression network analysis was performed based on all genes with reliable expression levels. Modules with similar eigengenes (MEs) were clustered (r > 0.75), resulting in a total of 13 co-expression modules. Among these, the turquoise, blue, and brown modules contained the largest number of genes, with 1255 (25.1%), 1226 (24.52%), and 1130 (22.6%) genes, respectively, while the salmon module contained the fewest genes (48, accounting for 0.96%). Correlation analysis between phenotypic traits and module eigengenes was conducted, and the correlation heatmap of each module was generated (Figure 5B). Among the 13 modules, five modules (brown, green-yellow, purple, tan, and yellow) showed positive correlations with the feeding group, of which only the yellow module was statistically significant (*p* < 0.05). Eight modules were positively correlated with the Control group, but only the blue module exhibited statistical significance (*p* < 0.05).

**Figure 6:**
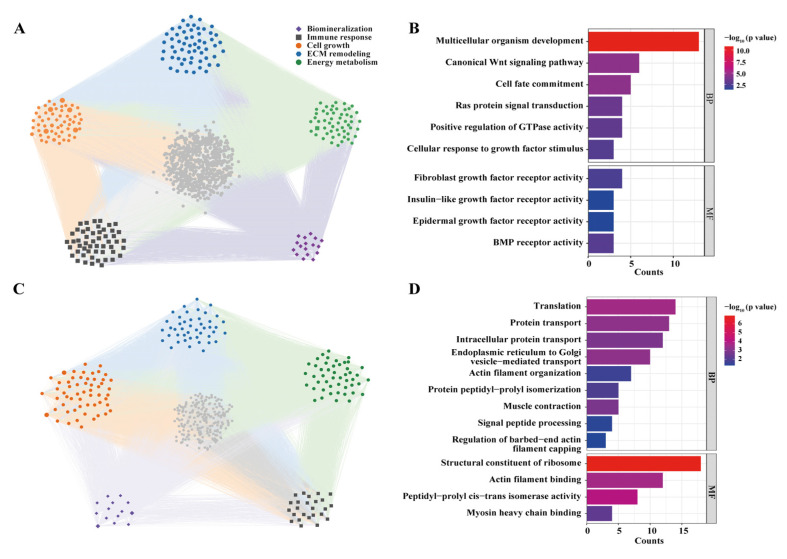
Co-expression module expression and functional enrichment analysis. (**A**) Gene co-expression network of the blue module. (**B**) GO enrichment analysis of genes in the blue module. (**C**) Gene co-expression network of the yellow module. (**D**) GO enrichment analysis of genes in the yellow module.

Subsequently, GO enrichment and network construction analyses were performed for these two significant modules. The genes in the blue module were significantly enriched in pathways associated with cell growth, energy metabolism, and biomineralization (*p* < 0.05), including multicellular organism development (GO:0007275), canonical Wnt signaling pathway (GO:0060070), cell fate commitment (GO:0045168), Ras protein signal transduction (GO:0007265), cellular response to growth factor stimulus (GO:0071363), fibroblast growth factor receptor activity (GO:0005007), epidermal growth factor receptor activity (GO:0005006), positive regulation of GTPase activity (GO:0043547), and BMP receptor activity (GO:0098821) (Figure 6A,B). The genes in the yellow module were extremely significantly enriched in translation (GO:0006412), endoplasmic reticulum to Golgi vesicle-mediated transport (GO:0006888), and protein transport (GO:0015031) (*p* < 0.001), and significantly enriched in intracellular protein transport (GO:0006886), muscle contraction (GO:0006936), protein peptidyl–prolyl isomerization (GO:0000413), actin filament organization (GO:0007015), signal peptide processing (GO:0006465), and regulation of barbed-end actin filament capping (GO:2000812) (*p* < 0.05). These enriched functions are closely related to cell growth and energy metabolism (Figure 6C,D).

## 4. Discussion

The coral reef ecosystems worldwide are facing severe degradation owing to bleaching, crown-of-thorns starfish outbreak and human activities. The coral reef restoration is the major way to compensate the loss of scleractinian corals, which requires huge amounts of coral donors. Our previous study demonstrated that some species of scleractinian corals are able to conduct polyp bailout and reattachment under environmental stress, which not only contributes to the living of coral communities but also offer a novel way to produce numerous coral colonies for coral reef restoration. In this study, it was proved that brine shrimp feeding could significantly accelerate the growth of reattached polyps of coral *P. damicornis* by enhancing energy reservation and immune capacity, which shed light on the fast and large-scale cultivation of coral donors for reef restoration.

Firstly, the influence of brine shrimp feeding on the growth of reattached polyps were observed. It was observed that reattached polyps of coral *P. damicornis* could capture and feed on brine shrimps. After feeding treatment for 60 days, the diameter of corals, numbers of new polyps, and the weight of calcified skeletons were all significantly increased, which reflected the obvious contribution of brine shrimp feeding to the growth of reattached coral polyps. Generally, the scleractinian corals acquire nutrients through mixotrophy [52]. They obtain autotrophic nutrition via symbiosis with Symbiodiniaceae, which transfer photosynthetic products to their coral hosts, while the coral-associated bacteria contribute to coral health through nitrogen fixation, pathogen defense, and the induction of larval settlement and metamorphosis [53,54]. Bacterial symbionts also contribute to nutrient cycling within the holobionts, though their roles are highly diverse and complex [55,56]. The reciprocal regulation of organic carbon availability for the coral host and inorganic nitrogen availability for the algal symbionts maintains a stable and balanced symbiosis, which is critical for the holobiont’s survival [57]. In addition, scleractinian corals can also obtain heterotrophic nutrition through the capture of organic particles in seawater. Corals with higher heterotrophic ability are able to sustain sufficient lipid reserve under stress [58,59], which is of great importance for their survival, because lipid reserve is required for stress response activities [52,60]. Thus, results from the present study suggest that reattached polyps of *P. damicornis* rely on mixotrophic nutrients, and heterotrophic nutrition contributes dramatically to the early growth and development of the juvenile corals by enhancing polyp formation and biomineralization. Feeding brine shrimp to the newly reattached polyps should be a novel and effective way to accelerate the growth of corals, which benefits the fast ana large-scale cultivation of coral donors for reef restoration. In addition, the heterotrophic plasticity is of great importance for corals since they rely on stronger heterotrophic activity to compensate for the energy gap caused by declined algal symbiont density or stress responses so as to sustain energy balance in the symbiotic association under environmental threats [61]. Thus, the current study indicates that the capturing of zooplankton, such as brine shrimps, will benefit the fast growth and adaptation of the reattached coral polyps in natural reef.

To further reveal the molecular mechanisms that brine shrimp feeding accelerates the growth of reattached coral polyps, parameters related to energy metabolism in corals, such as algal symbiont density, chlorophyll *a + c_2_* content, Ea, Ec and CEA, were tested in the present study. It was found that after 60 days of brine shrimp feeding, the symbiont density and chlorophyll *a + c_2_* content, as well as Ea in coral hosts and their algal symbionts, all elevated significantly. The symbiont density and chlorophyll *a + c_2_* content to some extend reflect the photosynthesis efficiency of the coral–Symbiodiniaceae holobiont, and coral productivity is primarily limited by ammonium availability [29,39]. According to recent study, the nitrogen environment and nitrogen status of the coral symbionts are important factors determining optimal photosynthetic carbon acquisition and hence holobiont growth. For example, overall carbon acquisition (per holobiont surface area) or per symbiont cell was enhanced by heterotrophic feeding (ammonium source) or direct ammonium supplementation, suggesting that nitrogen, not carbon, was limiting symbiont photosynthesis [62]. Results from the current study indicated that brine shrimp feeding might contribute to the elevation of symbiont density and chlorophyll *a + c_2_* content by enhancing ammonium supply, which subsequently benefited photosynthesis to accelerate the growth of holobionts. Moreover, to reveal the influence of brine shrimp feeding to the reattached coral polyps, parameters related to immune capacity, including T-AOC, MDA contents and caspase-3 activation level, were determined. It was demonstrated that the caspase-3 activation level in the coral hosts was significantly inhibited after brine shrimp feeding, and T-AOC in the feeding group was also slightly higher (no statistical difference) than that in the control group. In corals, T-AOC is closely related to antioxidant activity upon stress, which is important because excessive intracellular oxidative pressure can severely affect photosynthesis and activate the expression of caspase-3 to trigger apoptotic activities [39,56]. Corals with lower caspase-3 level (apoptotic rates) exhibit stronger immune capacity, which have higher survival chance under environmental threats. Collectively, results from the present study illustrate that brine shrimp feeding contributes to the growth of reattached polyps by elevating symbiont density and chlorophyll *a + c_2_* contents, and enhancing the immune capacity of corals to cope with environmental challenges.

**Figure 7:**
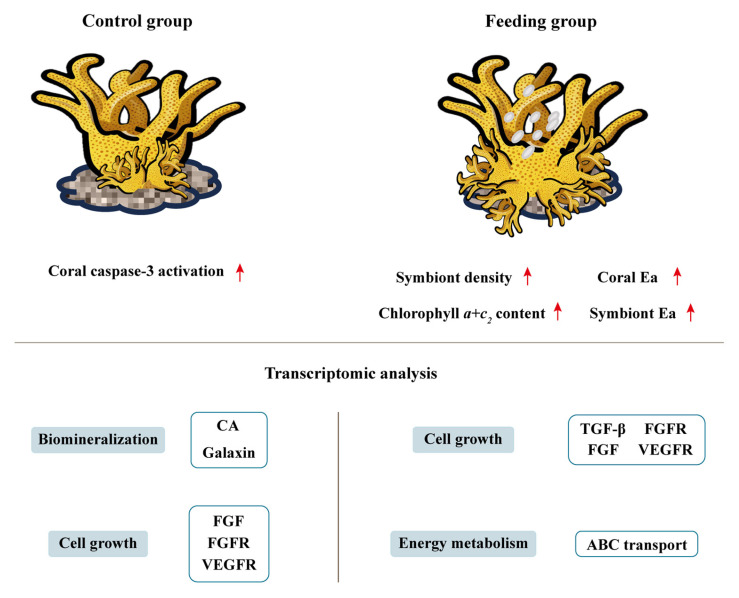
Physiological and transcriptomic differences between corals in control and feeding groups.

Furthermore, to in-depth explore the gene expression profiles of reattached polyps after brine shrimp feeding, corals were sampled for transcriptomic analysis on the 60th day post feeding treatment. GO terms related to growth and metabolism, multicellular organism development (GO:0007275), adenylate cyclase-activating G protein-coupled receptor signaling pathway (GO:0007189), positive regulation of kinase activity (GO:0033674), cell surface receptor protein tyrosine kinase signaling pathway (GO:0007169), and transmembrane receptor protein tyrosine kinase activity (GO:0004714) were enriched from the significantly up-regulated genes. Such results were in consistent with the elevated symbiont density and Ea values in the coral host, which represented the enhanced energy reservation. Moreover, results from GO enrichment and network construction analysis exhibited that the DEGs were significantly enriched in pathways associated with cell growth, energy metabolism, and biomineralization, including multicellular organism development (GO:0007275), Ras protein signal transduction (GO:0007265), cellular response to growth factor stimulus (GO:0071363), fibroblast growth factor receptor activity (GO:0005007), epidermal growth factor receptor activity (GO:0005006), and BMP receptor activity (GO:0098821). According to previous study, fibroblast growth factor (FGF) is present in the extracellular matrix, and when FGF binds to its receptor, it activates downstream Src family kinases and the Ras/Raf-MAPK pathway, which in turn promotes the secretion of matrix metalloproteinases (MMPs) [63]. In addition, bone morphogenetic proteins (BMPs) play important roles in body axes formation and tissue mineralization in both vertebrates and invertebrates. In coral, BMP2/4 is specifically labeled in the calicoblastic ectoderm, indicating that BMP2/4 may act as a signaling molecule of coral stem cells during biomineralization to ensure their differentiation into calcified cells [64]. Therefore, the current results infer that pathways related to cell growth, energy metabolism, and biomineralization were significantly activated after brine shrimp feeding, collectively contributing to the fast growth and high immune capability of the reattached polyps (Figure 7).

## 5. Conclusions

In summary, the present study illustrates that reattached polyps of coral *P. damicornis* can capture and feed on brine shrimps, which contributes significantly to their growth. The diameter, number of new polyps, weight of the calcified skeleton, symbiont density, chlorophyll *a + c_2_* content and Ea values of reattached coral polyps increased obviously, and signaling pathways responsive for energy metabolism, cell growth and biomineralization were dramatically activated. Moreover, brine shrimp feeding also enhances the immunity of the reattached polyps by suppressing apoptotic activity and elevating antioxidant capacity. These results reveal the influence and detailed molecular mechanisms of brine shrimp feeding on the growth of newly reattached coral polyps, which shed light on the potential application of such methods in the cultivation of coral transplantation donors.

There are inevitably obvious limitations in the current study since factors including species-specific feeding responses, water flow, predation on *Artemia* in outdoor systems, and operational feasibility, will all constrain the extension of conclusions drawn from laboratory-based experiments on a single species to ocean-scale restoration. In the future, more efforts are needed for exploring if feeding zooplankton like *Artemia* can benefit the initial growth of reattached coral polyps in natural reefs, and the potential application of such methods in reef restoration.

## Figures and Tables

**Table 1:** Gene expression mapping ratio.

Sample	Total Mapping Gene Ratio	Uniquely Mapping Gene Ratio
Control-1	41.35	39.53
Control-2	42.44	40.43
Control-3	42.3	40.46
Control-4	40.01	38.19
Control-5	40.93	39.12
Feeding-1	43.72	41.79
Feeding-2	38.62	36.87
Feeding-3	40.94	39.13
Feeding-4	42.49	40.61
Feeding-5	41.33	39.45

## Data Availability

The data that supports the findings of this study are available on request.
